# Amalgam

**Published:** 1877-08

**Authors:** 


### AMALGAM.

There seems to be a disposition on the part of some in the dental profession, during the past three or four years, to make a new (or an old) departure. This is in the direction of plastic material for filling teeth. Now this is not new; for the last thirty or forty years this "cry has been in the air." In nine cases in ten in which dentists have attempted to write on the desirability and importance of obtaining a plastic material of universal application, and one that should possess all the qualifications imaginable, hhey have simply settled down into a fulsome landation of amalgam, generally, or of some particular form of amalgam, and a condemnation of gold, at least in contrast with amalgam.

Some of these have a "new amalgam" of their own device, "better than anything else that ever was made," or can be made with such, "There are millions in it."

Usually gutta percha, and oxy. chloride of zinc are referred to as possessing some merit; but the burden of the song is amalgam, its efficiency, mode of use, etc. Almost every advocate says it is better than gold, and then he will usually, squarely contradicts himself before closing.

If a good and efficient amalgam for filling teeth is not found, it will not be for want of effort, or lack of experiment.

During the last thirty years there has been produced, upon an average, one new amalgam annually, each of which was better than all others, indeed, was about perfect, and so of course could not be superseded. It would seem that the formulas, so far as any distinctive character is concerned, have about been exhausted. But when we consider the various changes that may be made with four or five different materials, and the great variety of special modes of manipulation that may be employed with every one who desires a new formula, some special method of treatment and manipulation in preparing, is claimed as an important element.

Well care in this respect is not to be ignored, but the fact that alloys, for the most part, and amalgams always an exceedingly fickle in their behavior, under varying circumstances, has been overlooked by both those who prepare, and those who use amalgams for filling teeth. Dr. Fletcher has, perhaps, come nearer recognizing this peculiarity of alloys, than any one who has written upon the subject. He says: "An alloy used for amalgam, whatever its composition, is utterly unreliable, unless every ingot has been carefully tested for shrinkage, expansion, packing and retaining its shape under water and discoloration. However good an alloy may be generally, no single sample of it is trustworthy until after a series of tests which cannot be completed in less than three to six months." And he might have added that even after any given sample is thus tested, when it it comes to be combined with mercury, and subjected to varying outside influences, such as moisture, heat and electricity its behavior will lack uniformity in a greater or less degree, and Dr. Fletcher plainly asserts this fact where he says in the article quoted above:

"Unless moisture is totally excluded a soft amalgam is worthless, if both freedom from discoloration, and permanence are required."

In this brief article from which we quote, published in the June No. of the *Missouri Dental Journal*, Dr. Fletcher refers to some of the difficulties that attach to the use of amalgam as a material for filling teeth, and tries to give directions for avoiding them. Are they affective? Either the directions are very difficult to follow, or are sadly defective. We have seen many fillings made of Dr. Fletcher's amalgam, as signal failures, just as black and unsightly, notwithstanding "it will not discolor," and as leaky and worthless, as those made of a dozen other kinds of "perfect amalgams that will neither blacken nor shrink."

For the last twenty-five years every dentist who has devised a new formula for amalgam, has made the sterrætyped claim.

Free from oxydation; will not discolor; will not shrink, and will not admit moisture, will save the teeth, especially in all difficult cavities, better than gold, is easier used, and is less expensive to the patient, etc." Now the common experience of the profession is that these claims are extravagant, and present quite a deviation from the treth.

There is no amalgam made, so far as we are aware, that will not in some months oxydize, and there is none so base that in some months there will be no apparent oxydation

We know of no amalgam that will not sometimes contract, sufficient to admit moisture, and almost every one of which we have any knowledge will sometimes solidify without contraction, and exclude mositure, and of course such fillings may, and sometimes do prevent decay. There are many things that modify the various conditions in which amalgam fillings are found in the mouth, to these we may refer at another time.

